# To study the effect of oxygen carrying capacity on expressed changes of erythrocyte membrane protein in different storage times

**DOI:** 10.1042/BSR20200799

**Published:** 2020-06-25

**Authors:** Huan Wang, Han-Wei Wei, Hua-Chun Shen, Zhen-Zhou Li, Yong Cheng, Li-Shuang Duan, Lei Yin, Jun Yu, Jian-Rong Guo

**Affiliations:** 1 Department of Anesthesiology, Shanghai Gongli Hospital, The Second Military Medical University, Shanghai 200135, P.R. China; 2 HwaMei Hospital, University of Chinese Academy of Sciences, Zhejiang 315010, P.R. China; 3 Department of Anesthesiology, Yijishan Hospital, Wannan Medical College, Wuhu 241001, P.R. China; 4 Ningxia Medical University, Gongli Hospital of Shanghai Pudong New Area Training Base, Shanghai 200135, P.R. China

**Keywords:** erythrocyte, Membrane protein, oxygen carrying capacity, storage time

## Abstract

Erythrocyte membrane is crucial to maintain the stability of erythrocyte structure. The membrane protein on the surface of erythrocyte membrane enables erythrocyte to have plasticity and pass through the microcirculation without being blocked or destroyed. Decreased deformability of erythrocyte membrane protein will lead to a series of pathological and physiological changes such as tissue and organ ischemia and hypoxia. Therefore, this research collected 30 cases of healthy blood donors, and explored erythrocyte stored at different times relating indicators including effective oxygen uptake (Q), P50, 2,3-DPG, Na^+^-k^+^-ATP. Erythrocyte morphology was observed by electron microscopy. Western blot and immunofluorescence assay were used to detect membrane protein EPB41, S1P, GLTP, SPPL2A expression changes of erythrocyte. To explore the effective carry oxygen capacity of erythrocyte at different storage time resulting in the expression change of erythrocyte surface membrane protein.

## Introduction

Erythrocyte is the basic structural and functional substance to maintain life. As the main tangible component of blood, erythrocyte plays an important role in blood of exorcising the old and absorbing the new nutrient substance and gas. In the body, red blood cells are mainly responsible for transporting breathing gas, carrying oxygen inhaled from the lung to various tissues and organs of the body, and then transporting metabolite carbon dioxide from various parts, which is an indispensable transport team for the body [1].

**Figure 1:**
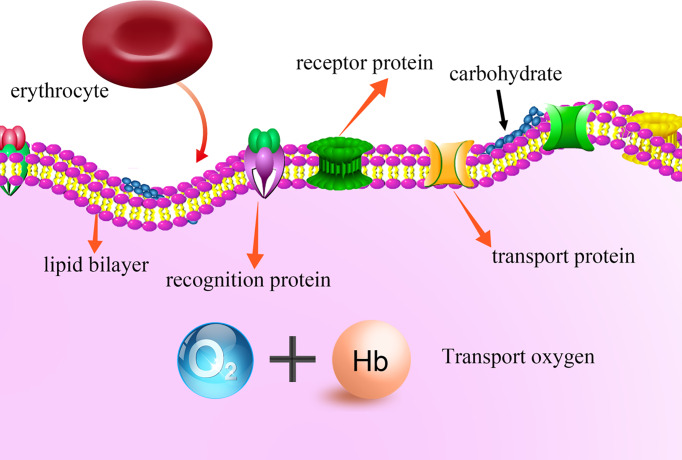
The structure of erythrocyte membrane

Normal erythrocytes are in a smooth shape of double concave disc, which makes erythrocyte can resist the shear stress of blood vessels and organ walls in the circulation of blood without breaking up. The framework protein system embedded on the surface of erythrocyte membrane can not only make cell membrane deformable, but also maintain the double-concave circular structure of the erythrocyte. The erythrocyte membrane maintains the stability of structure and resists external pressure to prevent its deformation. The major chemical components of erythrocyte membrane are lipids and some kinds of proteins, as shown in Figure 1. Human erythrocyte membrane contains ~49% protein, ~43% lipid, and ~8% carbohydrate. Lipids constitute the bilayer skeleton of cell membrane, cholesterol is inserted between phospholipids to enhance the stability of bilayer, proteins are irregularly embedded or floated in its surface, and can move freely in the membrane plane. The flow or contraction of membrane proteins make red blood cells be more plastic, which can pass through the microcirculation without being blocked or destroyed [2,3].

The unique flexibility and deformability of red blood cells are derived from their own membrane structure. According to the different functions of membrane proteins in the ultrastructure of red blood cells, membrane proteins can be divided into transport protein, recognition protein and receptor protein. Membrane proteins play a crucial role in the stability of erythrocyte structure and the normal physiological function [1]. The basic functional processes of erythrocyte, such as energy conversion, material transport, signal recognition and transduction, are closely related to the membrane protein of erythrocyte [4]. Erythrocyte surface protein EPB41 is a skeleton basic protein related to membrane protein of red blood cells, which plays a key role in normal cell formation and regulation of mechanical stability and variability of membrane physical properties [5]. In addition, S1P, GLTP, SPPL2A can participate in the reconstruction of erythrocyte membrane skeleton, changing the deformability, adhesion and membrane stability of cells, etc. [6]. Therefore, these membrane protein were selected to research the function of red blood cells. However, researches had shown that the structure and stability of erythrocyte membrane protein could change with the increase in preservation time [7,8]. Erythrocytes preserved for too long not only reduced the effective oxygen carrying capacity, but also affected the expression of erythrocyte membrane protein and the infusion effect of erythrocytes, even triggered immune hemolysis reaction [9]. Decreased deformability of erythrocyte membrane protein will lead to increased blood viscosity, increased circulatory resistance, reduced effective oxygen carrying capacity, and a series of pathological and physiological changes such as tissue and organ ischemia and hypoxia. Therefore, it is necessary to conduct more deep and extensive studies on the characteristics of changes in expression of erythrocyte surface membrane protein caused by differences in the effective oxygen carrying capacity of erythrocytes after different storage times, which will provide an important scientific basis for clinical application and evaluation of blood transfusion quality.

## Experiment

### Materials

Bottles of oxygen, carbon dioxide, nitrogen gas mixed gas path, stainless steel metal frame (Beijing Beipu Feilong Gas Co., Ltd.); flow meter (Changzhou Chengfeng Flow Meter Co., Ltd.); pressure gauge (Yangquan Precision Instrument Factory); Cy-3 digital oxygen analyzer (Shanghai Huaguang Instrument Factory); blood count instrument (bc-2800, Mindray); 37°C constant temperature water bath (GSY – II, Beijing Medical Equipment Factory); low-speed automatic balancing centrifuge B120 (Hebei Baiyang Centrifuge Plant); sampler (Eppendorf, 200 μl, 1000 μl); AB fresh/frozen plasma, defoaming agent (Beijing Huaruixiang Technology Co., Ltd.); 1 ml disposable syringe, 5 ml disposable test tube, disposable five-bag blood collection device (Sichuan Nanger Company); 2,3-Diphosphoglyceric acid (2,3-DPG) detection kit (Wuhan Huamei Biotechnology Co., Ltd.); Na^+^-k^+^-ATP assay kit (Nanjing Jiancheng Biological Co., Ltd.); 30 cases of blood donors who met the conditions for voluntary blood donation in China collected 1 unit of red blood cells (200 ml/U) each to prepare suspended red blood cells. Rabbit anti-Human primary antibody EPB41 (1:500), Rabbit anti-Human primary antibody S1P1 (1:500), Rabbit anti-Human primary antibody GLTP (1:500), Rabbit anti-Human primary antibody SPPL2A (1:500), Rabbit anti-Human primary antibody GADPH (1:1000) (Invitrogen, U.S.A.).

Inverted microscope (Nikon company), electrophoresis apparatus (Bio-Rad company), ImageQuant LAS4000mini chemiluminescence imager (GE company), NanoDrop2000 ultra-micro spectrophotometer (Thermo Scientific company), Nanosight nanoparticle tracking analyzer (Malvern Instruments, U.K.). Electron microscope (H7600 TEM, Hitachi, Japan), Getein 1100 Immunofluorescence Quantitative (Shanghai Feijie Biotechnology Co., Ltd, China).

### Methods

#### Preparation of suspension red blood cells

The whole blood was collected from 30 healthy blood donors, 400 ml for each case. Blood samples were tested for hepatitis b virus, hepatitis c virus, human immunodeficiency virus, treponema pallidum, and other transfusion-transmitted pathogens in accordance with the national blood and component blood quality requirements [10]. After passing the test, the blood was prepared into suspended red blood cells in strict accordance with the operation procedures of blood station technical operation regulations (2012 edition) [11]. Suspended red blood cells were served in five empty bags by using sterile bonder, and divided into 1 day group, 7 day group, 14 day group, 21 day group, 28 day group, respectively. Then they were stored in 2–6°C. Effective oxygen carrying capacity, P50, concentration of 2,3-DPG, and Na^+^-K^+^-ATP content in suspension red blood cells of each group were observed at 1, 7, 14, 21, and 28 days, respectively.

#### Determination of effective oxygen carrying capacity (Q) and P50 [12]

The whole blood was collected from 30 healthy blood donors, 400 ml for each case. The blood was centrifuged with 3300 rev/min at 4°C for 10 min after conventional anticoagulant treatment. Then removed plasma and added CPDA-1 erythrocyte preserve fluid to prepare suspended red blood cells. Suspended red blood cells coming from the same patient were divided into five copies and restored at 4°C. The five samples were tested in 1, 7, 14, 21, and 28 days, respectively. Simulation of arterial oxygen partial pressure, the test condition: O_2_ = 16 ml/min, the CO_2_ = 3 ml/min, N_2_ = 120 ml/min, flow rate: 100 ml/min, 37°C to sample inflatable 9 min. Finally, 1 ml of the sample was extracted for blood gas analysis. The charging conditions were adjusted as O_2_ = 6 ml/min, CO_2_ = 3 ml/min, N_2_ = 160 ml/min, and the flow rate was 100 ml/min. Balance for 10 min, 37°C to sample inflatable 6 min. Finally, 1 ml of the sample was also extracted for sexual blood gas analysis. According to the calculation formula of effective oxygen-carrying capacity of erythrocytes, *Q* = 20 × (*S*1 − *S*2), the unit was ml. P50 value was obtained from the blood gas analysis results when the oxygen partial pressure reached 100 mmHg.

#### Determination of 2,3-DPG and Na^+^-K^+^-ATP [13]

The whole blood was collected from 30 healthy donors, with each blood sample being 400 ml. The blood was treated by conventional anticoagulant and centrifuged for 10 min at 4°C with the speed of 3300 rev/min, then removed the plasma, erythrocyte preserve fluid (CPDA-1) was added into the suspended erythrocytes. Suspended erythrocytes will come from the same patient were divided into five portions, these five samples in the preservation of 1, 7, 14, 21, and 28 days were detected, respectively, according to the 2,3-DPG and Na^+^-K^+^-ATP kit instructions.

#### Observation of erythrocyte morphology

Five blood samples were collected from each person, and erythrocytes in different storage times 1, 7, 14, 21, and 28 days were collected, respectively. Then the erythrocytes were diluted to the appropriate concentration, smear was taken and the morphology was observed under scanning electron microscope (H7600 TEM, Hitachi, Japan).

#### Western blot analysis of EPB41, S1P, GLTP, and SPPL2A expression [14]

Total proteins of erythrocytes were extracted and 20 μg proteins were sampled. 5% concentrated gel and 12% isolated gel were prepared respectively to isolate proteins by SDS-PAGE. Objective and internal reference proteins were transferred to NC membrane, then closed with 5% skimmed milk powder sealing fluid for 2 h at room temperature. Rabbit anti-Human primary antibody EPB41(1:500), Rabbit anti-Human primary antibody S1P1 (1:500), Rabbit anti-Human primary antibody GLTP (1:500), Rabbit anti-Human primary antibody SPPL2A (1:500), Rabbit anti-Human primary antibody GADPH (1:1000) were added and incubated at 4°C overnight. TBST washed four times, then HRP labeled Sheep anti-Rabbit secondary antibody (1:5000) was added and incubated at 37°C for 1 h. TBST washed four times. Color was developed with ECL luminescent solution, protein bands were exposed by gel image analysis system, and images were photographed and quantitatively analyzed. The experiment was repeated three times.

#### Immunofluorescence analysis of EPB41, S1P, GLTP, and SPPL2A expression [15]

The separated and purified erythrocytes were added into Eppendorf tube (No. 1 and 2). 500 μl 2% paraformaldehyde and 500 μl 0.0075% glutaraldehyde were added into the cells, shook them slowly, leaf them at room temperature for 30 min. The mixture was centrifuged with 900 rev/min for 2 min, and discarded the upper organic phase. 1 ml PBS was added to two tubes, and the supernatant was discarded after repeated centrifugation. The protein occlusive solution (5% BSA) was added to the cells (tubes 1 and 2) treated in the previous step, shook well and sealed at room temperature for 40 min. Rabbit anti-Human primary antibody EPB41 (1:500), Rabbit anti-Human primary antibody S1P1 (1:500), Rabbit anti-Human primary antibody GLTP (1:500), Rabbit anti-Human primary antibody SPPL2A (1:500), Rabbit anti-Human primary antibody GADPH (1:1000) were added to tube 1. Common solution IgG antibodies were added to tube 2, gently shook well, and incubated at 4°C overnight. The upper primary antibody solution was removed after centrifuging with 3700 rev/min for 6 min. Secondary antibodies (Goat anti-Rabbit, isothiocyanate fluorescein FITC marker) were added into tube 1 and tube 2 for incubation for 1 h, and the whole operation process of secondary antibodies should be strictly protected from light. Red blood cells were centrifuged and the upper fluid was discarded after centrifuging with 3700 rev/min for 6 min. 800 μl PBS buffer was added and repeated centrifugation for four to five times to wash the remaining unbound primary and secondary antibodies. The erythrocytes were suspended in 100 μl PBS buffer for smear observation.

### Statistical analysis

All experiments were replicated independently at least three times. The data were analyzed using one-way analysis of variance (ANOVA) and are presented as the mean ± standard deviation (SD). Statistical significance was defined as *P*<0.05.

## Results and discussion

### Identification of oxygen carrying capacity

**Figure 2:**
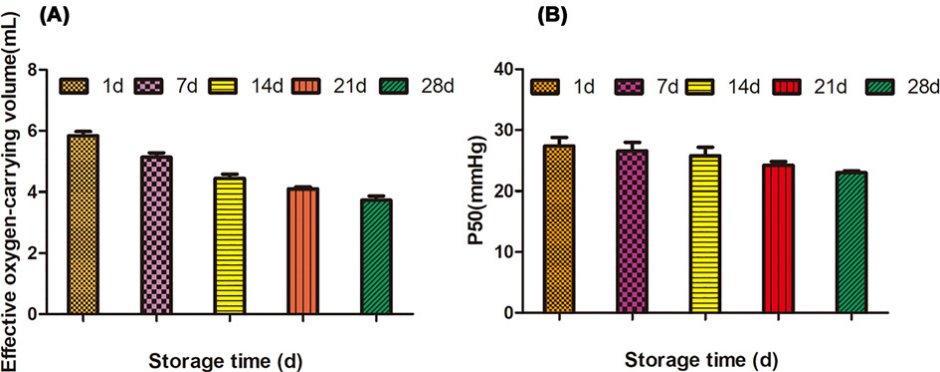
The changes of carrying oxygen capacity in suspension RBC (**A**) Effective oxygen-carrying volume; (**B**) P50 of suspension RBC.

The results of effective oxygen carrying capacity (Q) and P50 are shown in Table 1. It could be seen from Table 1 that the effective oxygen carrying capacity in red blood cell suspension decreased gradually with the increase in storage time. Figure 2A showed that the effective oxygen carrying capacity decreased by 24.0% at the storage of 14 day and 36.0% at the storage of 28 day. Figure 2B showed that the P50 of red blood cell suspension decreased by 6.86% at the storage of 14 day, and 9.74% at the storage of 28 day, but there was no significant difference (*P*>0.05).

**Table 1:** Changes of red blood cells oxygen carrying capacity at different storage time

	(days)	1	7	14	21	28
Suspension RBC	Q (ml)	5.83±0.20	5.13±0.20	4.43±0.20	4.10±0.08	3.73±0.21
	P50 (mmHg)	27.7±2.01	26.7±2.01	25.8±2.01	24.2±0.81	25.0±2.01

The morphology and function of erythrocyte are closely related to its oxygen carrying and releasing ability. The carrying process of oxygen and carbon dioxide in human body mainly depends on red blood cells. The blood transfusion method is commonly used in medical emergency to increase the blood circulation of patients, which can improve the oxygen carrying and releasing ability of blood [16]. The transport of oxygen in the body all depends on the affinity of hemoglobin with oxygen. In the case of high partial pressure of oxygen, oxygen is easy to combine with hemoglobin, while in the case of low partial pressure of oxygen, oxygen and hemoglobin are easily dissociated [17]. It can be seen from Figure 2A that the oxygen partial pressure of red blood cells decreased gradually with the extension of storage time, which indicated that the effective oxygen carrying and oxygen releasing capacity of red blood cells decreased.

P50 is defined as partial oxygen pressure at a blood oxygen saturation of 50%, and the value of P50 indicates that the affinity of red blood cells with oxygen in the blood [18]. As shown in Figure 2B, the affinity of red blood cells with oxygen gradually decreased as the storage time increased. The value of P50 is inversely proportional to affinity of erythrocyte with oxygen. The larger P50 is, the smaller oxygen affinity of erythrocyte is, the weaker ability combined with oxygen is, and the easier releasing oxygen is. On the contrary, the smaller P50 is, the stronger binding capacity of erythrocyte is, and the harder releasing oxygen is [19]. Analysis of erythrocyte effective oxygen carrying and releasing capacity (Q) and erythrocyte oxygen affinity (P50), the storage time of erythrocyte is too long, which will affect its corresponding physiological function. Higher P50 can deliver oxygen to hypoxic tissues more quickly when used in transfusion emergency treatment, which has great clinical significance.

### Determination of 2,3-DPG and Na^+^-K^+^-ATP

**Figure 3:**
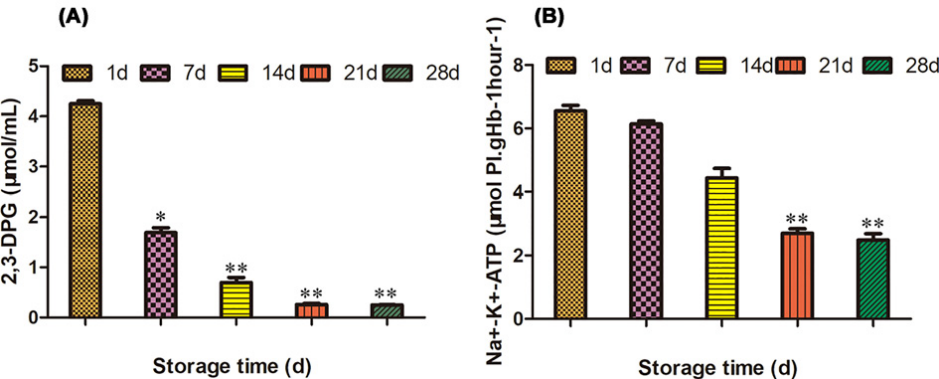
The changes of 2,3-DPG, Na^+^-K^+^-ATP in red blood cells at different storage times (**A**) The concentration of 2,3-DPG; (**B**) the content of Na^+^-K^+^-ATP. 2,3-DPG. *P<0.05; **P<0.01.

The concentration of 2,3-DPG in red blood cells decreased 60.23% on the 7th day, 83.53% on the 14th day, 93.88% on the 21st day, and 94.11% on the 28th day (Table 2). It could be seen that after red blood cells being stored for one month, the concentration of 2,3-DPG basically disappeared (Figure 3A). The concentration of Na^+^-K^+^-ATP in red blood cells decreased gently before 7 days and only decreased 6.26% on the 7th day, but sharply decreased 32.37% on the 14th day, 58.77% on the 21st day, and 62.0% on the 28th day (Figure 3B).

**Table 2:** Changes of erythrocyte oxygen carrying capacity at different storage time

(days)	1	7	14	21	28
2,3-DPG (μmol/ml)	4.25±0.08	1.69±0.13	0.70±0.14	0.26±0.03	0.25±0.01
Na^+^-K^+^-ATP (μmol PI/gHb/h)	6.55±0.25	6.14±0.13	4.43±0.43	2.70±0.20	2.49±0.28

2,3-DPG is an important index to evaluate the oxygen carrying capacity of red blood cells. It can specifically bind to hemoglobin (Hb) to reduce the oxygen affinity of hemoglobin, there by regulating the release of hemoglobin oxygen [20]. Without the presence of 2,3-DPG in red blood cells, hemoglobin shows a high affinity for oxygen. Meanwhile, oxygen affinity (P50) is reduced, so it cannot effectively carry oxygen. Therefore, the higher the concentration of 2,3-DPG is, the better effective oxygen carrying capacity of red blood cells is. When the concentration of 2,3-DPG increasing, the affinity between Hb and oxygen decreases and oxygen is easily released. When the concentration of 2,3-DPG decreases, the affinity of Hb to oxygen increases, which is not conducive to oxygen release [21].

Red blood cells mainly regulates the content of intracellular Na^+^ and K^+^ to adjust the volume of the cell. These cationic depended on Na^+^-K^+^-ATP on the membrane, which plays an important role in maintaining normal cell morphology and providing ATP for cells survive [22]. The decreased activity of Na^+^-K^+^-ATP reduces the release of ATP energy and affects the normal metabolism of red blood cells. Therefore, Na^+^-K^+^-ATP is also an important indicator to evaluate the function of red blood cells. The results of our study showed that the decrease in Na^+^-K^+^-ATP was the most significant before 14 days of storage, with a decline of 32.37%. The decrease in Na^+^-K^+^-ATP caused the release of K^+^ in the cells and the imbalance between the internal and external ions in the red blood cells, thus affecting the oxygen carrying capacity of the red blood cells [23].

### Observation of erythrocyte morphology

**Figure 4:**
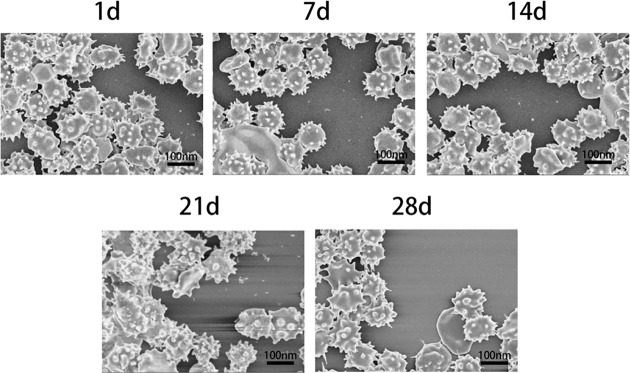
The change of erythrocyte morphological in different storage time

Electron microscopy was used to observe the morphological changes of erythrocytes storing for 1, 7, 14, 21, and 28 days. In the first 2 weeks, it could be seen from the electron micrograph that it was presented spinous globular erythrocytes. The micrograph of erythrocytes at 21th day showed that cells were significantly sparser than those before 21 day, and even more sparse and irregular when they stored at 28th day. As it could be seen in Figure 4, the red blood cells have distinct divot-disk conformation characteristics and uniform size (5–8 μm). Erythrocytes play an important role in improving microcirculation disorders and cerebral ischemia. In addition, normal and healthy erythrocyte morphology plays an important role in improving microcirculation disorders, cerebral ischemia and other clinical diseases.

### Western blot analysis of EPB41, S1P, GLTP, and SPPL2A expression

**Figure 5:**
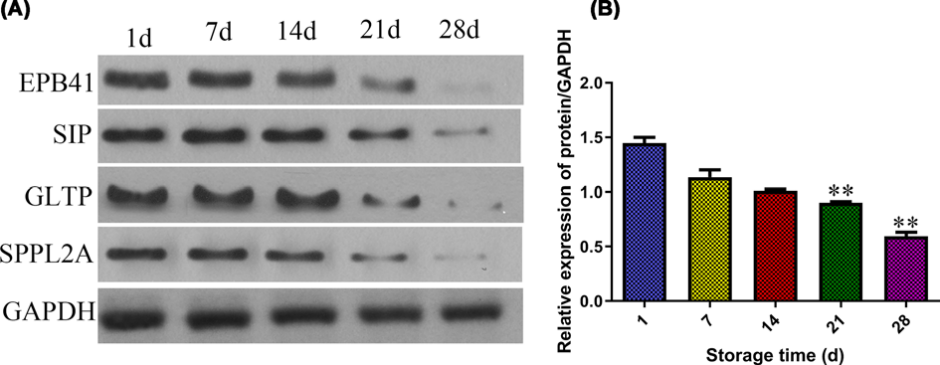
Protein level of MRPS35 in red blood cells in different storage times determined by Western blotting Mean ± SD, *n* = 3. **P<0.01 vs 7 days group.

The expression of erythrocyte surface membrane proteins EPB41, S1P, GLTP, and SPPL2A were detected by Western blot. As shown in Figure 5, protein EPB41, S1P, GLTP, and SPPL2A all decreased with the increase in erythrocyte storage time. There was no significant difference in the first 21 days (*P*>0.05), while the results of protein expression existed significant difference on the 28th day comparing with the 7th day (**P<0.01).

Erythrocyte must have extraordinary deformability and stability, which are derived from the unique membrane protein molecular composition of the erythrocyte membrane and the network structure formed by the combination of these molecules [24], and play a decisive role in maintaining structural integrity of the membrane. Both the absence and abnormal expression of erythrocyte membrane protein can cause severe erythrocythemia, which eventually leads to hemolytic anemia [25,26]. Previous studies had shown that the structure and stability of erythrocyte membrane protein would change with the increase in preservation time [27]. Erythrocytes have no nucleus and lack physiological structure of inner membrane system. Changes in membrane proteins are also important signaling molecules of erythrocyte senescence clearance. Loss and structural changes of these proteins are observed during preservation, which may lead to membrane structure changes and thereby affect infusion effect [28,29].

### Immunofluorescence analysis of EPB41, S1P, GLTP, and SPPL2A expression

**Figure 6:**
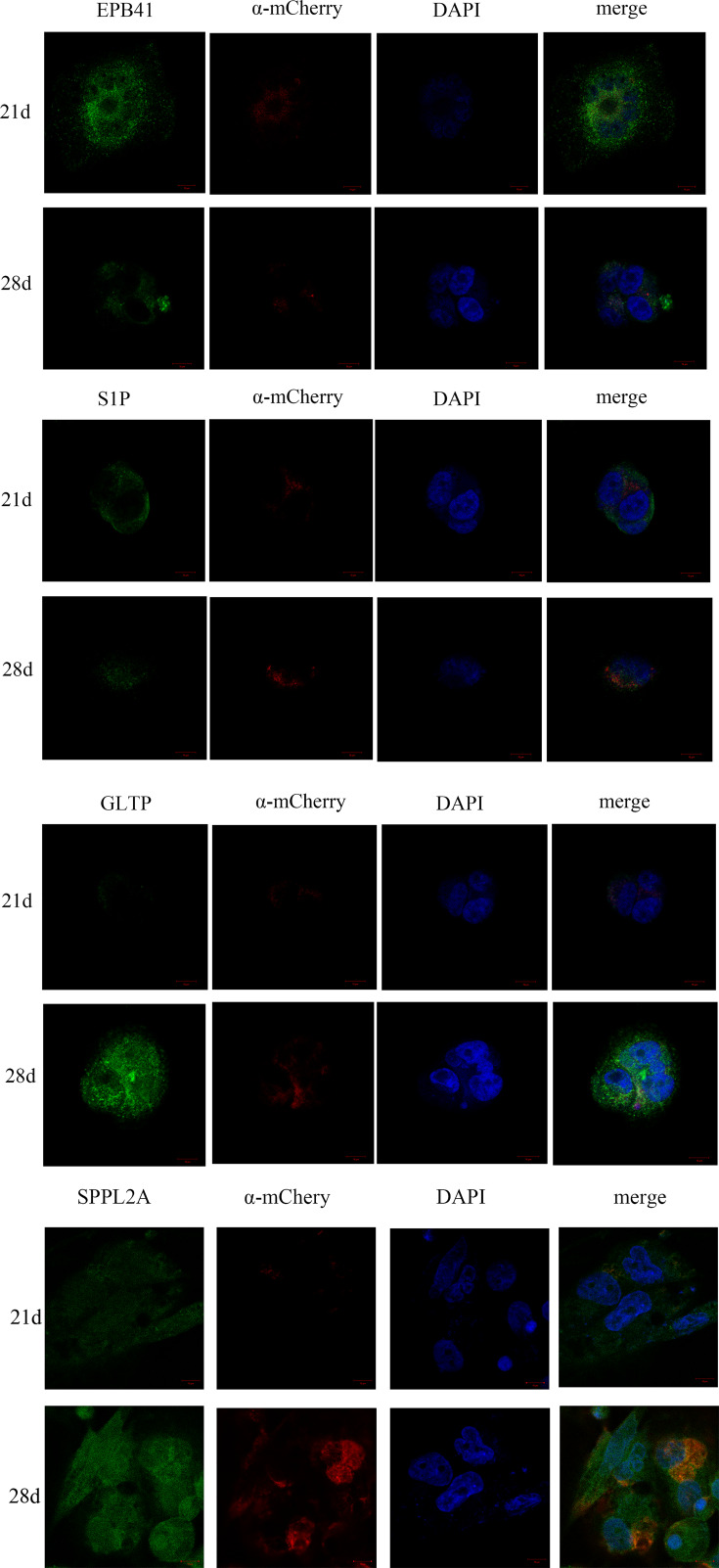
Immunofluorescence analysis of erythrocyte surface membrane protein EPB41, S1P, GLTP

Membrane proteins EPB41, S1P, GLTP, and SPPL2 of erythrocyte were stained by FITC, and observed by laser confocal microscope. As shown in the Figure 6, the number and activity of EPB41 and S1P protein in 21 days were greater than that in 28 days, which indicated that the expression of membrane protein decreased with the increase in erythrocyte storage time. It could be clearly seen from Figure 6 that a large number of red blood cells were stained with fluorescent signals and observed in the bright field. The green fluorescence was brighter at the cell edge and darker in the middle. The bright fluorescence signals on the cell membrane were the result of concentrated fluorescence staining of membrane proteins with dense distribution. The target membrane protein could be analyzed *in situ* by means of immunofluorescence. the expression and activity of GLTP protein in 21 days were lower than that in 28 days, GLTP can be used to increase glycolipid levels over natural levels in either side of the membrane leaflet [30].

In a word, changes in lipids and proteins on the cytomembrane will affect the normal physiological functions of the membrane. Diseases are closely related to cytomembrane lipids and proteins. Some researches suggested erythrocyte membrane fluidity was significantly reduced, which was affected by lipid metabolism and other factors [4]. Therefore, a thorough understanding of the components, structure, and interactions of erythrocyte membrane proteins is particularly important for the rational selection of erythrocyte storage time. However, there are still some disadvantages, such as the small sample size and the limited geographical source of patients, which need a further study.

## Conclusion

Erythrocyte membrane is crucial to maintain the stability of erythrocyte structure. The membrane protein EPB41, S1P, GLTP, SPPL2A expression on the surface of erythrocyte membrane were decreased after storing at different times, which would affect the effective carry oxygen capacity of erythrocyte.

## Abbreviations

2,3-DPG: 2,3-diphosphoglyceric acid
SD: standard deviation
